# Effects of clonidine on microcirculatory alterations during endotoxemia

**DOI:** 10.1186/cc12326

**Published:** 2013-03-19

**Authors:** K Schmidt, C Philipsenburg, A Zivkovic, S Hofer

**Affiliations:** 1 Universitätsklinikum Heidelberg, Germany Critical

## Introduction

Endothelial dysfunction during endotoxemia is responsible for the functional breakdown of microvascular perfusion and microvessel permeability. The cholinergic anti-inflammatory pathway (CAP) is a neurophysiological mechanism that regulates the inflammatory response by inhibiting proinflammatory cytokine synthesis, thereby preventing tissue damage. Endotoxemia-induced microcirculatory dysfunction can be reduced by cholinergic CAP activation. Clonidine improves survival in experimental sepsis [1] by reducing the sympathetic tone, resulting in the parasympathetic-mediated CAP activation. The aim of this study was to determine the effects of clonidine on microcirculatory alterations during endotoxemia.

## Methods

Using fluorescent intravital microscopy, we determined the venular wall shear rate, macromolecular efflux and leukocyte adhesion in mesenteric postcapillary venules of male Wistar rats. Endotoxemia was induced over 120 minutes by intravenous infusion of lipopolysaccharide (LPS). Control groups received an equivalent volume of saline. Clonidine 10 μg/kg was applied as i.v. bolus in treatment groups. Animals received either (i) saline alone, (ii) clonidine 10 minutes prior to saline administration, (iii) clonidine 45 minutes prior to LPS administration, (iv) clonidine 10 minutes prior to LPS administration, (v) clonidine 30 minutes after LPS administration or (vi) LPS alone.

## Results

All LPS groups (iii to vi) showed a significantly reduced venular wall shear rate compared with the saline group after 120 minutes. There were no significant differences between the numbers of adhering leukocytes in the clonidine/LPS groups (iii, iv, v) and the LPS group after 120 minutes. Macromolecular efflux significantly increased in all groups over the time period of 120 minutes. After 120 minutes there was no difference between the LPS group and the clonidine 10 minutes prior to LPS administration group (iv) whereas all other groups (i, ii, iii, v) showed a significantly reduced macromolecular efflux compared with the LPS group.

## Conclusion

Clonidine has no positive effect on microhemodynamic alterations and leukocyte-endothelial interaction during endotoxemia. The reduction of capillary leakage in clonidine-treated groups depends on the time interval relative to the initiation of endotoxemia. Endothelial permeability and leukocyte activation are regulated by different pathways when stimulated by clonidine during endotoxemia. We conclude that clonidine might have an important time-dependent anti-inflammatory and protective effect on endothelial activation during inflammation.

## References

[B1] Hofer S Crit Care 2009 13 R11 10.1186/cc7709 19196475 PMC2688128

